# Clinical Outcomes of Oral Suspension versus Delayed-Release Tablet Formulations of Posaconazole for Prophylaxis of Invasive Fungal Infections

**DOI:** 10.1128/AAC.00893-18

**Published:** 2018-09-24

**Authors:** Jon P. Furuno, Gregory B. Tallman, Brie N. Noble, Joseph S. Bubalo, Graeme N. Forrest, James S. Lewis, Ana F. Bienvenida, Courtney A. Holmes, Bo R. Weber, Jessina C. McGregor

**Affiliations:** a Department of Pharmacy Practice, Oregon State University/Oregon Health & Science University College of Pharmacy, Portland, Oregon, USA; b Department of Pharmacy Services, Oregon Health & Science University Hospitals and Clinics, Portland, Oregon, USA; c Division of Infectious Diseases, VA Portland Healthcare System, Portland, Oregon, USA

**Keywords:** antifungal agents, formulation, invasive fungal infection, medical outcomes, posaconazole, prophylaxis

## Abstract

Posaconazole is used for prophylaxis for invasive fungal infections (IFIs) among patients with hematologic malignancies. We compared the incidence of breakthrough IFIs and early discontinuation between patients receiving delayed-release tablet and oral suspension formulations of posaconazole. This was a retrospective cohort study of patients receiving posaconazole between 1 January 2010 and 30 June 2016. We defined probable or proven breakthrough IFIs using the European Organization for Research and Treatment of Cancer (EORTC) criteria. Overall, 547 patients received 860 courses of posaconazole (53% received the oral suspension and 48% received the tablet); primary indications for prophylaxis were acute myeloid leukemia (69%), graft-versus-host disease (18%), and myelodysplastic syndrome (3%). There were no significant differences in demographics or indications between patients receiving the different formulations. The incidence and incidence rate of probable or proven IFIs were 1.6% and 3.2 per 10,000 posaconazole days, respectively. There was no significant difference in the rate of IFIs between suspension courses (2.8 per 10,000 posaconazole days) and tablet courses (3.7 per 10,000 posaconazole days) (rate ratio = 0.8, 95% confidence interval [CI] = 0.3 to 2.3). Of the 14 proven or probable cases of IFI, 8/14 had posaconazole serum concentrations measured, and the concentrations in 7/8 were above 0.7 μg/ml. Posaconazole was discontinued early in 15.5% of courses; however, the frequency of discontinuation was also not significantly different between the tablet (16.5%) and oral suspension (14.6%) formulations (95% CI for difference = −0.13 to 0.06). In conclusion, the incidence of breakthrough IFIs was low among patients receiving posaconazole prophylaxis and not significantly different between patients receiving the tablet formulation and those receiving the oral suspension formulation.

## Abstract (precis)

Posaconazole is used for prophylaxis for invasive fungal infections (IFIs) among patients with hematologic malignancies. We compared the incidence of breakthrough IFIs and early discontinuation between patients receiving delayed-release tablet and oral suspension formulations of posaconazole.

## INTRODUCTION

Invasive fungal infections (IFIs) are associated with considerable excess morbidity, mortality, and costs among infected patients. Immunocompromised patients, including patients with hematologic malignancies receiving hematopoietic stem cell transplants and immunosuppressive therapy, are at increased risk of IFIs and associated poor outcomes (1–3). Posaconazole prophylaxis has been recommended to prevent IFIs and improve patient outcomes in these high-risk patients (4, 5). A randomized controlled trial concluded that posaconazole more effectively prevented IFIs and was associated with lower all-cause mortality than fluconazole or itraconazole among neutropenic patients (6).

The suboptimal absorption of the oral suspension formulation of posaconazole and the requirements of the dosing regimen may have limited its effectiveness. A delayed-release tablet formulation of posaconazole was approved in December 2013, and previous studies have suggested that this formulation improves absorption and bioavailability (7, 8). However, improved clinical outcomes with the tablet formulation compared to the outcomes with the oral suspension have not been clearly demonstrated. In this study, we expand upon prior work performed at Oregon Health & Science University Hospitals and Clinics (OHSU) to assess the clinical outcomes between the oral suspension and tablet formulations of posaconazole for prophylaxis for IFIs. We also identified and compared the frequency and rationale for discontinuation of posaconazole and postdiscontinuation outcomes between patients receiving the different formulations of posaconazole prophylaxis.

(These data were presented in part at ASM Microbe 2017, June 2017, New Orleans, LA, and IDWeek 2017, October 2017, San Diego, CA.)

## RESULTS

**FIG 1:**
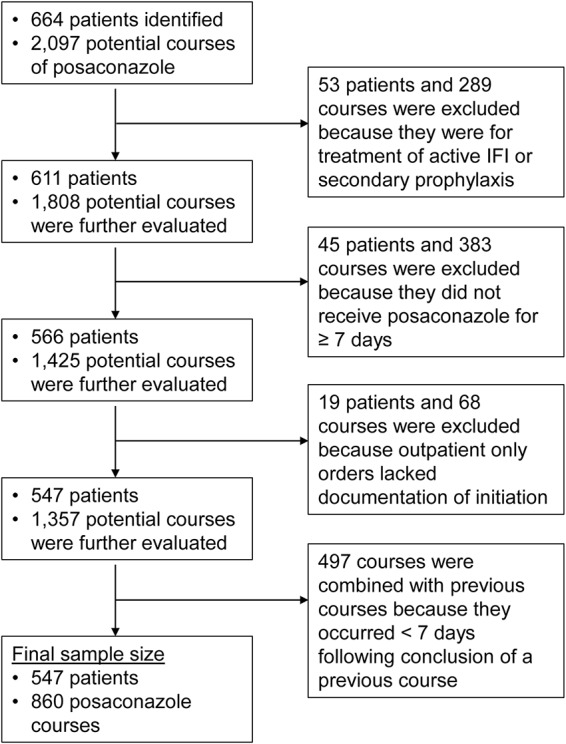
Flowchart of study cohort derivation.

We initially identified 664 patients, which represented 2,097 potential courses of posaconazole, for possible inclusion in this study. However, following application of our exclusion criteria (Fig. 1), our final sample size was 547 patients representing 860 courses of posaconazole, of which 452 courses (52.6%) were the oral suspension formulation and 408 courses (48.4%) were the tablet formulation. Included patients were more frequently male (61.8%) and had a median Charlson comorbidity index of 3 (interquartile range [IQR], 2 to 5) (data not shown). The course-level characteristics of the patients stratified by posaconazole formulation are displayed in Table 1. Patients who received the tablet formulation were more likely to be greater than 65 years old than patients who received suspension courses (27.7% versus 21.9%, *P* = 0.049). Additionally, patients who received oral suspension courses were more likely to receive nasogastric tube administration of posaconazole than patients who received the tablet formulation (2.9% versus 0.7%, *P* = 0.02). The median duration of prophylaxis was similar between the two formulations (median, 24 days [IQR, 14 to 54.5 days] for the oral suspension versus 25 days [IQR, 15 to 49 days] for the tablets, *P* = 0.93). Acute myeloid leukemia (AML) was the most prevalent indication for receiving posaconazole prophylaxis, and its incidence was similar between patients receiving suspension courses and patients receiving tablet courses (67.5% versus 69.9%, *P* = 0.19). Patients receiving suspension posaconazole courses were significantly more likely than patients receiving tablet courses to have nasogastric tube administration (2.9% versus 0.7%, *P* = 0.02) and a diagnosis of mucositis during their course (15.7% versus 8.9%, *P* = 0.002). In contrast, patients receiving posaconazole suspension courses were less likely than patients receiving tablet courses to have posaconazole serum concentrations measured (46.5% versus 67.6%, *P* < 0.001). Among those with serum concentration measurements, patients receiving suspension posaconazole courses were less likely to have achieved a target level of ≥0.7 mg/liter (60.5% versus 90.6%, *P* < 0.001). There was no significant difference in the Charlson comorbidity index between formulations in both groups (median, 3; IQR, 2 to 5; *P* = 0.34).

**TABLE 1:** Characteristics of posaconazole courses^*a*^

Characteristic	Value for patients receiving:			*P*
	Total (*n* = 860)	Oral suspension (*n* = 452)	Tablet (*n* = 408)	
No. (%) of courses among patients aged >65 yr	212 (24.7)	99 (21.9)	113 (27.7)	0.049
No. (%) of courses among patients with BMI of >30 kg/m^2^	280 (32.6)	153 (33.9)	127 (31.1)	0.40
Median (IQR) Charlson comorbidity index	3 (2–5)	3 (2–5)	3 (2–5)	0.34
Median (IQR) duration of prophylaxis (days)	25 (15–52)	24 (14–54.5)	25 (15–49)	0.93
No. (%) of courses among patients with nasogastric tube administration	16 (1.9)	13 (2.9)	3 (0.7)	0.02
No. (%) of courses among patients with the following prophylaxis indication:				
Acute myeloid leukemia	590 (68.6)	305 (67.5)	285 (69.9)	0.19
Graft-vs-host disease	155 (18.0)	84 (18.6)	71 (17.4)	
Myelodysplastic syndrome	27 (3.1)	14 (3.1)	13 (3.2)	
Acute lymphoblastic leukemia	25 (2.9)	15 (3.3)	10 (2.5)	
Aplastic anemia	17 (2.0)	10 (2.2)	7 (1.7)	
Other high-dose steroid use	12 (1.4)	3 (0.7)	9 (2.2)	
Other leukemia	12 (1.4)	10 (2.2)	2 (0.5)	
Post-allogeneic hematopoietic stem cell transplant	12 (1.4)	4 (0.9)	8 (2.0)	
Chronic myeloid leukemia	4 (0.5)	4 (0.9)	0 (0.0)	
Chronic lymphocytic leukemia	2 (0.2)	1 (0.2)	1 (0.3)	
Multiple myeloma	2 (0.2)	1 (0.2)	1 (0.3)	
Other	2 (0.2)	1 (0.2)	1 (0.3)	
Met NCCN criteria for prophylaxis	754 (87.7)	390 (86.3)	364 (89.2)	0.14
Mucositis during course	106 (12.3)	71 (15.7)	35 (8.9)	0.002
No. (%) of courses among patients receiving the following other medications:				
Antiviral therapy	641 (74.5)	338 (74.8)	303 (74.3)	0.86
Antibiotic therapy	764 (88.8)	413 (91.4)	351 (86.0)	0.01
Proton pump inhibitors	457 (53.1)	250 (55.3)	207 (50.7)	0.18
No. (%) of courses among patients:				
In whom the posaconazole serum concn was measured during the course	486 (56.5)	210 (46.5)	276 (67.6)	<0.001
Who achieved the target serum concn (≥0.7 mg/liter) (*n* = 486)	377 (77.6)	127 (60.5)	250 (90.6)	<0.001
No. (%) of courses among patients with the following chemotherapy regimen (*n* = 566):				0.48
Cytarabine + idarubicin ± methotrexate	198 (35.0)	103 (35.4)	95 (34.5)	
Cytarabine ± methotrexate	144 (25.4)	78 (26.8)	66 (24.0)	
Cytarabine + idarubicin + fludarabine ± methotrexate	46 (8.1)	22 (7.6)	24 (8.7)	
Cytarabine + mitoxantrone + etoposide ± methotrexate	34 (6.0)	15 (5.2)	19 (6.9)	
Other	23 (4.1)	14 (4.8)	9 (3.3)	
Azacitidine	22 (3.9)	11 (3.8)	11 (4.0)	
Cytarabine + mitoxantrone ± methotrexate	19 (3.4)	8 (2.7)	11 (4.0)	
Cytarabine + fludarabine	13 (2.3)	7 (2.4)	6 (2.2)	
Cytarabine + daunorubicin	12 (2.1)	4 (1.4)	8 (2.9)	
Cytarabine + idarubicin + mitoxantrone + etoposide	11 (1.9)	6 (2.1)	5 (1.8)	
Azacitidine + cytarabine + idarubicin ± methotrexate	10 (1.8)	5 (1.7)	5 (1.8)	
Methotrexate	10 (1.8)	6 (2.1)	4 (1.5)	
Cytarabine + idarubicin + tretinoin ± methotrexate	8 (1.4)	3 (1.0)	5 (1.8)	
Fludarabine	5 (0.9)	2 (0.7)	3 (1.1)	
Idarubicin + tretinoin	5 (0.9)	5 (1.7)	0 (0.0)	
Cytarabine + etoposide	2 (0.4)	0 (0.0)	2 (0.7)	
Fludarabine + cytarabine + mitoxantrone + etoposide	2 (0.4)	0 (0.0)	2 (0.7)	
Fludarabine + cytarabine + mitoxantrone + etoposide + methotrexate + idarubicin	2 (0.4)	2 (0.7)	0 (0.0)	

a Data are for 860 courses. BMI, body mass index; IQR, interquartile range; NCCN, National Comprehensive Cancer Network.

We identified 57 patients to be candidates for having an IFI that were subsequently reviewed by the expert panel. Following review by our clinical expert panel, 14 were determined to have a probable or proven IFI. Specifically, there were 3 probable and 4 proven cases of IFIs among patients receiving the suspension and 1 probable and 6 proven cases among patients receiving the tablet formulation. The incidence and incidence rate of probable or proven IFIs were 1.6% and 3.2 cases per 10,000 posaconazole days (95% confidence interval [CI] = 1.8 to 5.4 cases per 10,000 posaconazole days), respectively. There was no significant difference in the rate of IFIs between suspension courses (2.8 per 10,000 posaconazole days) and tablet courses (3.7 per 10,000 posaconazole days) (rate ratio [RR] = 0.8, 95% CI = 0.3 to 2.3). Results from unadjusted and adjusted Cox proportional hazards regression models of the effect of formulation on the risk of breakthrough IFIs are shown in Table 2. Tablet courses were more likely to be associated with breakthrough IFIs than suspension courses (adjusted hazard ratio [HR] = 1.2, 95% CI = 0.4 to 3.4), although this difference was not statistically significant. Nasogastric tube administration was significantly associated with an increased likelihood of breakthrough IFIs (adjusted HR = 34.1, 95% CI = 6.3 to 128.9); however, only 16 (1.9%) courses had nasogastric tube administration. Additionally, an age of >65 years (adjusted HR = 3.7, 95% CI = 1.2 to 12.1) and mucositis (adjusted HR = 4.6, 95% CI = 1.0 to 17.5) were associated with a statistically significantly increased likelihood of breakthrough IFIs. Results from the sensitivity analysis including only the first course of posaconazole prophylaxis per patient (*n* = 547) were not meaningfully different from results including all posaconazole courses; i.e., the estimates from both models were similar in direction and magnitude (data not shown).

**TABLE 2:** Adjusted and unadjusted hazard of breakthrough IFI among patients receiving posaconazole prophylaxis^*a*^

Characteristic	Unadjusted HR (95% CI)	Adjusted HR (95% CI)
Tablet formulation	1.1 (0.4–3.1)	1.2 (0.4–3.4)
Nasogastric tube administration	23.9 (4.6–83.4)	34.1 (6.3–128.9)
Male sex	2.0 (0.7–7.8)	2.2 (0.7–8.6)
Antiviral therapy	0.4 (0.1–1.1)	0.4 (0.1–1.2)
Underlying diagnosis (cancer vs GVHD and other high-dose steroid use)	1.5 (0.5–5.9)	
Antibiotic therapy	0.9 (0.2–8.5)	
Proton pump inhibitors	0.5 (0.2–1.4)	
Mucositis	2.5 (0.6–7.5)	4.6 (1.0–17.5)
Met NCCN criteria for prophylaxis	1.3 (0.3–12.0)	
Posaconazole level taken during course	1.0 (0.4–2.8)	
Age > 65 years	2.6 (0.9–7.1)	3.7 (1.2–12.1)
BMI > 30 (kg/m^2^)	0.6 (0.2–1.9)	

a Data are for 860 courses. GVHD, graft-versus-host disease; NCCN, National Comprehensive Cancer Network; BMI, body mass index.

A description of the 14 cases of breakthrough IFIs is shown in Table 3. The majority of cases were classified as proven IFIs (71.4%), and the most prevalent site of infection was the lung (50%). The median length of prophylaxis prior to breakthrough IFIs was 16 days (IQR, 13 to 40 days). Only 8/14 (57%) cases had posaconazole serum concentrations measured during their course, of which 7 (87.5%) reached the target concentration of ≥0.7 mg/liter. Antifungal susceptibility data were available for 4 of the 10 cases of proven IFIs. MICs of 0.5 μg/ml (Candida tropicalis) and 1 μg/ml (Candida glabrata) were observed for the two tested yeast isolates, and MICs of 1 μg/ml (*Mucor* spp.) and >16 μg/ml (Fusarium proliferatum) were observed for the two tested molds.

**TABLE 3:** Characteristics of patients who had probable or proven invasive fungal infections while receiving posaconazole prophylaxis^*a*^

Patient	Age >65 yr	Sex	Underlying diagnosis	HSCT	Posaconazole formulation	EORTC classification	Probable criteria	Site of infection	Identified fungal organism	No. of days after start of prophylaxis	Posaconazole level (μg/ml)
1	Yes	F	APL	No	Suspension	Probable	Host factor, neutropenia; clinical criteria, tracheobronchial plaques on bronchoscopy; mycological criteria, Aspergillus fumigatus from bronchoalveolar lavage fluid	Lung/trachea	Aspergillus fumigatus	15	NC
2	No	F	CML	No	Suspension	Probable	Host factor, neutropenia; clinical criteria, CT of the chest with multiple pulmonary nodules and focal peribronchial consolidation; mycological criteria, positive galactomannan	Lung	Positive galactomannan only	18	0.28
3	Yes	M	AML	No	Suspension	Proven		Skin	Fusarium proliferatum	17	1.82
4	Yes	M	AML	No	Tablet	Probable	Host factor, neutropenia; clinical criteria, CT with numerous bilateral small consolidative nodules; mycological criteria, positive galactomannan	Lung	Positive galactomannan only	25	1.80
5	Yes	M	AML	No	Tablet	Proven		Lung	Positive cryptococcal antigen only	13	NC
6	No	M	GVHD	Allogeneic (2004)	Suspension	Proven		Blood	Candida glabrata	12	1.07
7	No	M	AML	No	Tablet	Proven		Sinuses	*Mucor* sp.	66	0.90
8	Yes	M	AML	Allogeneic (2013)	Tablet	Proven		Sinuses	Zygomyces sp.	13	NC
9	No	M	AML	No	Suspension	Proven		Lung	Aspergillus niger	5	NC
10	No	M	AML	No	Suspension	Probable	Host factor, neutropenia; clinical criteria, multifocal bilateral inflammatory nodularity and consolidation compatible with multifocal pneumonia; mycological criteria, positive galactomannan	Lung	Positive galactomannan only	7	NC
11	No	F	Allogeneic HSCT	Allogeneic (2012)	Suspension	Proven		Lung	Aspergillus sp.	44	2.09
12	No	M	GVHD	Allogeneic (2014)	Tablet	Proven		Blood	Candida glabrata	78	1.20
13	No	M	AML	Allogeneic (2012)	Tablet	Proven		Sinuses	No culture growth	39	NC
14	Yes	M	AML	No	Tablet	Proven		Blood	Candida tropicalis	13	1.60

a Data are for 14 patients. F, female; M, male; HSCT, hematopoietic stem cell transplantation; EORTC, European Organization for Research and Treatment of Cancer; CT, computed tomography; APL, acute promyelocytic leukemia; CML, chronic myeloid leukemia; AML, acute myeloid leukemia; GVHD, graft-versus-host disease; NC, not collected.

The frequency and rationale for the early discontinuation of posaconazole prophylaxis among courses that met NCCN criteria for prophylaxis are shown in Table 4. Posaconazole prophylaxis was discontinued early in 15.5% of the courses; suspension courses were less likely to be discontinued early than tablet courses, but the difference was not statistically significant (14.6% versus 16.5%; 95% CI for difference, −0.13 to 0.06). The most frequent reasons for early discontinuation were elevated liver function tests (27.8%), an inability to take the oral formulation (20.9%), and cost (18.3%). Additionally, 14 patients discontinued prophylaxis as part of changes in goals of care but were not included as early discontinuations. There were no statistically significant differences in the early discontinuation rationale between suspension courses and tablet courses (*P* = 0.26). Among the 115 patients who had early the discontinuation of posaconazole prophylaxis, 27 (23.5%) had at least one additional course of posaconazole prophylaxis after early discontinuation.

**TABLE 4:** Frequency and rationale for early discontinuation of posaconazole prophylaxis among patients who met the National Comprehensive Cancer Network criteria

Characteristic	No. (%) of courses			*P*
	Total (*n* = 741)	Oral suspension (*n* = 384)	Tablet (*n* = 357)	
Discontinuation	115 (15.5)	56 (14.6)	59 (16.5)	0.47
Rationale				0.26
Elevated liver function tests	32 (27.8)	13 (23.2)	19 (32.2)	
Inability to take oral formulation	24 (20.9)	10 (17.9)	14 (23.7)	
Cost	21 (18.3)	8 (14.3)	13 (22.0)	
Low levels/poor absorption	10 (8.7)	6 (10.7)	4 (6.8)	
Tolerability^*a*^	10 (8.7)	6 (10.7)	4 (6.8)	
Drug shortage	3 (2.6)	3 (5.4)	0 (0.0)	
Drug interaction	3 (2.6)	3 (5.4)	0 (0.0)	
QT prolongation	2 (1.7)	1 (1.8)	1 (1.7)	
Unknown	10 (8.7)	6 (10.7)	4 (6.8)	

a Tolerability included patient-reported symptoms (e.g., nausea) sufficient to warrant discontinuation.

The observed mortality rate while receiving posaconazole for patients receiving the oral suspension formulation was 1.8 per 10,000 posaconazole days, and that for patients receiving the tablet formulation was 1.1 per 10,000 posaconazole days (RR = 1.1, 95% CI = 0.2 to 9.6).

## DISCUSSION

In this large, retrospective cohort study of patients receiving posaconazole prophylaxis, the incidence of breakthrough IFIs was low and not significantly different between patients receiving the tablet and those receiving the oral suspension formulation. Posaconazole prophylaxis was discontinued while still indicated in 17% of courses; however, the frequency of discontinuation was also not significantly different between the two formulations. The primary reasons for early discontinuation were elevated liver function tests, an inability to take an oral formulation, and drug cost. These data support the suggestion that breakthrough IFIs are rare among patients receiving posaconazole prophylaxis and that safety and effectiveness endpoints are similar between the tablet and oral suspension formulations.

To our knowledge, only three previous studies have compared the incidence of breakthrough IFIs between the oral suspension and tablet formulations of posaconazole (9–11). One study was of 63 pediatric patients with hematologic malignancies in Germany in which no IFIs were identified (10). The second included 152 patients with AML or myelodysplastic syndrome (MDS), and the authors reported an overall IFI incidence of 7% but did not observe a significant difference between the two formulations (9). However, breakthrough IFIs were a secondary endpoint, and the study was not powered to assess differences regarding this outcome. Furthermore, 4/8 cases of IFIs were possible IFIs rather than probable or proven IFIs. Subtracting these patients from the outcome group results in an incidence of 3.5%, which is closer to the 1.6% observed in our study and consistent with data from randomized controlled trials of posaconazole prophylaxis (6, 12). The most recent study included only 61 patients, with only 1 patient experiencing an IFI (11).

Among the 14 cases of proven or probable IFIs in our study, 8 cases had documented posaconazole serum concentration measurements, and the concentrations in 7 of these patients were above the proposed therapeutic level of 0.7 μg/ml. This proposed target level is based on limited evidence which was derived from a secondary analysis of two phase 3 clinical trials which demonstrated an exposure-response relationship between posaconazole levels and clinical failure (13). However, that analysis utilized a composite endpoint of clinical failure that was not aligned with the primary endpoint in either of the phase 3 trials contributing data (14). Additional studies also support the exposure-response relationship for both prophylaxis and treatment with recommended target levels ranging from 0.5 to 1.25 μg/ml, although these studies were limited by small sample sizes and a lack of antifungal susceptibility data (15–18). Our study and others support the superiority of the tablet over the suspension in achieving target serum concentrations (8, 11, 19). Our finding that IFIs occurred despite achieving these targets has been observed in a recent cohort study and also supports a reexamination of the proposed target concentration, the association between serum and infection site concentrations, and the value of routine monitoring for achievement of the current target (20). However, we also recognize the relative rarity of IFIs among patients receiving posaconazole prophylaxis, the associated costs of routine monitoring, and the fact that other factors beyond pharmacokinetic and/or pharmacodynamic properties contribute to the incidence of breakthrough IFIs.

We observed that 17% of patients discontinued posaconazole despite a still present indication for prophylaxis based on NCCN criteria. The primary documented rationales for early discontinuation were elevated liver function tests, an inability to take an oral formulation, and drug cost. There were no significant differences in the frequency or distribution of the rationale for early discontinuation between formulations, which is consistent with the findings reported from previous studies (9, 21). Evaluation of the clinical outcomes of patients that discontinued posaconazole was beyond the scope of this study, but they should be explored to better understand the relative trade-offs between those outcomes and the rationale for discontinuation.

Given that this was an observational study, the posaconazole formulation was not randomly assigned and thus is subject to potential confounding by indication. However, the impact of this bias is likely minimal given the temporal preference for the different formulations; i.e., once the tablet formulation was available, nearly all prescribers ceased using the suspension. Furthermore, the only significant differences between oral suspension and tablet courses were that oral suspension courses were more to likely to be administered using a nasogastric tube, the patients receiving oral suspension courses also received antibiotic therapy, and the patients receiving oral suspension courses experienced mucositis during their posaconazole course. Previous studies have also reported minimal differences in patient characteristics between patients who received the different formulations, and these differences were unlikely to influence our clinical outcomes (9, 22). After adjusting for formulation and course characteristics, nasogastric tube administration was associated with breakthrough IFIs; this may be because of the strong correlation with disease severity or suboptimal infection site drug concentrations. Regarding the difference in mucositis incidence between the two formulations, our study was not designed to explore this association. Chemotherapy is known to cause mucositis; however, there was no significant difference in the distribution of chemotherapeutic agents between patients receiving the two formulations. The only other study to quantify this association also observed a higher incidence of mucositis among patients who received the oral suspension formulation; however, that difference was not statistically significant (9).

The primary limitation of this study was that our retrospective study design required reliance on medical record documentation for determination of IFIs and the frequency and rationale for posaconazole discontinuation. As such, there is some potential for misclassification of these outcomes. In addition, our study was limited in its ability to address temporal changes in antifungal resistance or the incidence of IFIs. Temporal changes and a lack of randomization may also contribute to the observation that, while the difference was small (<1/10,000 posaconazole days) and not statistically significant, the incidence of IFIs was higher among patients who received the tablet formulation. Lastly, despite this being the largest cohort study of patients receiving posaconazole prophylaxis, the results of this single-site study may not be generalizable to other centers and patient populations, where practice patterns and the prevalence of antifungal resistance may vary.

In conclusion, we observed no significant difference in breakthrough IFIs between patients receiving oral suspension and tablet formulations of posaconazole prophylaxis, despite the significantly higher frequency of target level attainment among patients receiving the tablet formulation. Further work is needed to better identify target posaconazole levels, particularly in the context of increasing antifungal resistance.

## MATERIALS AND METHODS

### Study design and patient population.

This was a retrospective cohort study of patients with a hematologic malignancy who received posaconazole prophylaxis for IFIs as either an inpatient or an outpatient at Oregon Health & Science University Hospitals and Clinics (OHSU) between 1 January 2010 and 30 June 2016. This study was approved by the OHSU institutional review board. OHSU is located in the Portland, OR, metropolitan area and includes a 576-bed academic, tertiary referral center with the Knight Cancer Institute, which performs over 150 hematopoietic stem cell transplants a year, with outpatient care being provided in the Center for Hematologic Malignancies of the Knight Cancer Institute, an NCI-designated Comprehensive Cancer Center. Consistent with clinical guidelines, IFI prophylaxis with posaconazole was provided during the study period for patients with neutropenia secondary to treatment of acute myeloid leukemia (AML) or myelodysplastic syndrome (MDS), an allogeneic stem cell transplant, or treatment with high-dose steroids (>20 mg/day prednisone or equivalent) or when clinically indicated at the discretion of the physician (4, 23). At OHSU, the oral suspension of posaconazole was utilized for prophylaxis beginning in 2008 and was changed to the delayed-release tablet formulation in February 2014. After 1 February 2014, only 36 courses of the oral suspension were prescribed, and no courses of the oral suspension were prescribed after October 2015.

To be included in this analysis, patients must have received a minimum of 7 continuous days of posaconazole prophylaxis during the study period to ensure that drug serum concentrations were at steady state. Patients may have received multiple courses of posaconazole prophylaxis during the study period, and separate courses were defined as occurring more than 7 days after the end of the previous course. Patients could contribute multiple courses to the analysis until they had a probable or proven IFI.

### Data sources.

The primary data source for this study was the Pharmacy Research Repository (PHARR), a longitudinal repository of patient health care data created in partnership with the Oregon Clinical and Translational Institute (OCTRI) Research Data Warehouse (RDW) at OHSU. PHARR includes encounter, diagnosis, procedure, laboratory, and pharmacy data. For all study subjects, administrative, demographic, diagnosis, laboratory, and pharmacy data were extracted from that index encounter as well as all past and future encounters (inpatient and outpatient) within any OHSU setting. These data have been validated and used in previous epidemiologic studies of medication utilization and treatment outcomes (24, 25). In addition to these electronic data, we manually reviewed the medical records of all study patients to screen for candidate IFIs and confirm posaconazole start and stop dates to determine unique courses. We also confirmed the indication for posaconazole prophylaxis and the incidence and rationale for early discontinuation through medical record review. All manually extracted data were entered into a secure database using the Research Data Electronic Data Capture (REDCap) application and merged with electronically collected data from PHARR (26).

### Variable definitions.

Our primary exposure of interest was the formulation of posaconazole used for prophylaxis (i.e., either the delayed-release tablet or the oral suspension). The drug formulation was determined using pharmacy orders and the medication administration record. Prophylaxis (versus active treatment) was determined using a combination of duration of therapy, dosage, and frequency and confirmed using microbiology, timing, diagnosis data, and documentation in the medical record to rule out posaconazole treatment of proven or probable IFIs.

Our primary outcome of interest was breakthrough IFIs, which were defined as IFIs occurring while the patient was receiving posaconazole prophylaxis. Patients with candidate IFIs were identified during medical record review by changes in antifungal therapy or addition of an additional antifungal agent(s), positive fungal biomarkers (e.g., Aspergillus galactomannan, 16S rRNA sequencing results), or clinician documentation of an IFI or a suspicion of an IFI. All patients with a candidate IFI were reviewed by a clinical expert panel consisting of an infectious disease pharmacist (J.S.L.), oncology pharmacist (J.S.B.), and infectious disease physician (G.N.F.) to identify proven or probable IFIs based on European Organization for Research and Treatment of Cancer (EORTC) definitions (27). Two reviewers were randomly selected to independently review each patient. If there was disagreement, a third reviewer broke ties.

Our secondary outcome of interest was the discontinuation of posaconazole while it was still indicated; i.e., patients still met the National Comprehensive Cancer Network (NCCN) criteria for prophylaxis. Patients who did not initially meet the NCCN criteria were not evaluated for early discontinuation. Early discontinuation was defined using pharmacy orders and the medication administration record to identify initiation of a new systemic antifungal therapy and posaconazole discontinuations that occurred during the expected duration of prophylaxis. We then manually reviewed each medical record to determine the reason for early discontinuation. If the rationale for discontinuation was not explicitly stated in the medical record, two external reviewers (J.S.B., J.S.L., or G.N.F.) reviewed each patient, and ties were broken as described above for determination of IFIs.

We also collected data on other patient characteristics of interest, including demographics (e.g., age, sex), indications for IFI prophylaxis, the presence of comorbid illnesses and neutropenia, trough levels of posaconazole, and other medications that the patients were receiving (e.g., chemotherapy regimen, antibiotics, antiviral therapy, proton pump inhibitors) (23). Indications for prophylaxis and comorbid illnesses were defined using encounter diagnosis codes (International Classification of Diseases, versions 9 [ICD-9] and 10 [ICD-10]) from encounter and problem list diagnoses.

### Statistical analysis.

We used descriptive statistics, including means and standard deviations (SDs), medians and interquartile ranges (IQRs), and proportions, to summarize the characteristics of the patients and the treatment courses of the oral suspension and tablet formulations of posaconazole. The characteristics of the patients and treatment courses were compared between formulations using two-sample *t* tests, chi-squared tests, Fisher's exact test, and the Wilcoxon rank sum test as appropriate. We assessed within-subject correlations since patients could have multiple treatment courses. We calculated the observed rate of IFIs for each formulation along with the rate ratio (RR) and the exact 95% confidence intervals (CIs) using the conditional maximum likelihood estimate.

We performed Cox proportional hazards modeling to account for various lengths of follow-up and to adjust for potential confounders when estimating the effect of the posaconazole formulation on breakthrough IFIs. Since the occurrence of IFIs was rare, we used penalized Cox proportional hazards regression using Firth's correction to account for the high percentage of censoring (i.e., monotone likelihood). We calculated unadjusted and adjusted hazard ratios (HRs) and the corresponding 95% penalized profile-likelihood CIs. Variables were selected for inclusion in the model using Akaike information criterion (AIC) optimization within best subset selections with the posaconazole formulation forced into the model. To identify if associations remained consistent within the subset of the patients' first course of posaconazole, a sensitivity analysis was performed.

We compared the frequency of the early discontinuation of posaconazole and the documented reason for discontinuation between the two formulations using the chi-squared test. We also calculated the rate of death within 30 days after the conclusion of posaconazole prophylaxis and death while receiving posaconazole for each formulation, in addition to the RR and exact 95% confidence intervals, using the conditional maximum likelihood estimate. *P* values of less than 0.05 were considered statistically significant. All statistical analyses were performed using SAS statistical software, version 9.2 (SAS Institute, Cary, NC).
